# Association Between Digestive Power and Serum Vitamin B12 Levels in Young Adult Females: An Analytical Cross-Sectional Study

**DOI:** 10.7759/cureus.115427

**Published:** 2026-08-30

**Authors:** Pallavi Turkar, Kiran Tawalare, Aparna Karnad, Ajeet Saoji, Vijay Kirkan, Sakshi Dawale, Chinmayee Kadwe, Nikhil Kajale

**Affiliations:** 1 Department of Kriya Sharir, Shri Ayurved Mahavidyalaya, Nagpur, IND; 2 Department of Community Medicine, N.K.P. Salve Institute of Medical Sciences and Research Centre, Nagpur, IND

**Keywords:** ayurveda, digestive metabolism, digestive power (agni), digestive strength, micronutrient deficiency, nutrient absorption, nutritional status, vitamin b12

## Abstract

Background: Digestive power (Agni) is a fundamental Ayurvedic concept that governs digestion and metabolism. Vitamin B12 is an essential micronutrient required for red blood cell formation, neurological function, and DNA synthesis. However, scientific evidence evaluating the relationship between digestive power and vitamin B12 levels remains limited. This study aimed to determine the association between digestive power and serum vitamin B12 levels among females aged 19-28 years using a laboratory immunoassay method.

Materials and methods: A cross-sectional analytical study was carried out among 94 young adult females, including 47 vitamin B12-deficient and 47 vitamin B12-nondeficient females aged 19-28 years, selected through convenience sampling. Serum vitamin B12 was estimated using a standard laboratory immunoassay method, and participants were classified into four categories based on digestive strength (Agnibala) using a validated assessment tool. Data were analyzed using descriptive statistics, Pearson's chi-square test, independent Student's t-test, and multiple linear regression. A p-value ≤ 0.05 was considered statistically significant.

Results: Serum vitamin B12 levels differed significantly between the deficient and nondeficient groups (p < 0.001). Balanced digestive power (Samagni) was predominantly observed among vitamin B12-nondeficient participants. In contrast, irregular digestive power (Vishamagni), elevated digestive power (Tikshnagni), and subdued digestive power (Mandagni) were more common among vitamin B12-deficient participants. The distribution of digestive power was significantly associated with vitamin B12 status, as demonstrated by Pearson's Chi-square test (χ² = 23.16, df = 3, p < 0.001).

Conclusion: Digestive power showed a significant association with serum vitamin B12 levels. Balanced digestive power was predominantly observed in individuals with normal vitamin B12 levels, whereas disturbed digestive power was relatively higher in deficient participants. These findings suggest that digestive metabolic efficiency may be associated with micronutrient assimilation and warrant further prospective studies.

## Introduction

Globally, vitamin B12 deficiency is becoming more common, especially among vegetarian populations. Vitamin B12 is a water-soluble vitamin essential for neurological function, red blood cell formation, and DNA synthesis. Its deficiency may lead to a wide range of physiological and systemic abnormalities [1-3]. Vitamin B12 deficiency commonly results from impaired absorption and may present with anaemia, fatigue, dizziness, palpitations, peripheral neuropathy, depression, and cognitive impairment [4,5].

In Ayurveda, digestive power (Agni) is considered the fundamental principle governing digestion and metabolism and is regarded as essential for maintaining vital essence (Oja), metabolic energy (Teja), vital life force (Prana), vitality, complexion, strength, and overall health [6]. Among the different types of Agni, central digestive fire (Jatharagni) is regarded as the principal digestive force responsible for digestion and metabolism. Based on its functional capacity, Jatharagni is classified into four categories: balanced digestive power (Samagni), irregular digestive power (Vishamagni), elevated digestive power (Tikshnagni), and subdued digestive power (Mandagni) [7]. Digestive strength (Agnibala), assessed using a validated self-assessment questionnaire, reflects the functional status of an individual's digestive power and was used in the present study to categorize participants into these four digestive power states.

According to Ayurvedic principles, disturbed digestive power is believed to impair nutrient assimilation and may be associated with vitamin B12 deficiency, metabolic imbalance, and lifestyle-related disorders [8]. Therefore, investigating the association between digestive power and serum vitamin B12 levels may provide valuable insight into the relationship between digestive function and vitamin B12 status. Accordingly, the present study was undertaken to investigate the association between Agnibala and serum vitamin B12 levels among young adult females.

## Materials and methods

Study design and setting

This cross-sectional analytical study was conducted and reported in accordance with the Strengthening the Reporting of Observational Studies in Epidemiology (STROBE) guidelines at Shri Ayurved Mahavidyalaya, Nagpur, Maharashtra, India [9]. Participant recruitment, assessment, and data collection were performed after obtaining approval from the Institutional Ethics Committee.

Study duration

The study was conducted from 08 October 2024 to 04 January 2026. Participant recruitment, questionnaire-based assessment, and serum vitamin B12 estimation were completed during this period. Statistical analysis was performed after completion of data collection.

Selection criteria

The study included only vegetarian females aged 19-28 years because animal-derived foods are the principal dietary source of vitamin B12​​​​​​, and dietary intake is a major determinant of vitamin B12 status. Restricting the study population to vegetarians minimized dietary confounding and enabled a more reliable assessment of the association between digestive power and serum vitamin B12 levels. Furthermore, limiting the study to females within a defined age range reduced biological variability related to sex and age, thereby improving the internal validity of the study.

Sample size calculation

The sample size was estimated using the standard formula for estimating a single population proportion based on the prevalence of vitamin B12 deficiency reported in previous literature. 

$n = \frac{Z^2 \times p \times (1-p)}{d^2}$

Where n is the required sample size, Z is the standard normal deviate at a 95% confidence level (1.96), p is the prevalence of vitamin B12 deficiency (0.143), and d is the absolute precision (0.10). A prevalence of 14.3% reported in a previous study was used for sample size estimation [10].

Accordingly,

$n = \frac{(1.96)^2 \times 0.143 \times 0.857}{(0.10)^2}$

*n = 47.1 ≈ 47*

Thus, the minimum required sample size was estimated to be 47 participants.

Since the primary objective of the study was to compare Agnibala between vitamin B12-deficient and vitamin B12-nondeficient participants, an equal number of participants were enrolled in each group. Accordingly, the final study population comprised 94 young adult females, including 47 vitamin B12-deficient and 47 vitamin B12-nondeficient participants. Eligible participants were recruited using convenience sampling until the required sample size for each group was achieved.

Serum vitamin B12 status was determined using a standard laboratory immunoassay method. Participants with serum vitamin B12 levels below 160 pg/mL were classified as vitamin B12 deficient, whereas those with serum vitamin B12 levels within the laboratory reference range (160-900 pg/mL) were classified as vitamin B12-nondeficient [10,11].

Data collection and assessment

Agnibala was assessed using the validated self-assessment questionnaire developed by Singh et al. [12]. Permission to use the questionnaire was obtained from the original copyright holders prior to publication of the study.

The questionnaire comprises 11 self-administered items, each containing four response options corresponding to the four classical Agni categories: Samagni, Vishamagni, Tikshnagni and Mandagni. Participants completed the questionnaire independently under the supervision of the investigators, and the completed questionnaires were reviewed for completeness before scoring according to the validated method. For each participant, responses corresponding to each digestive power category were summed separately and converted into percentage scores using the validated scoring method:

$\text{Percentage score} = \frac{\text{Score obtained for a particular digestive power category}}{\text{Maximum possible score for that category}} \times 100$

As specified in the original validated questionnaire, the maximum obtainable score was 11 for subdued, irregular, and balanced digestive power, and 10 for elevated digestive power, because one questionnaire item did not include a response option for elevated digestive power. The digestive power category with the highest percentage score was considered the participant's dominant digestive power and was used for participant classification and subsequent statistical analysis.

Balanced digestive power represents normal and efficient digestion under physiological conditions. Irregular digestive power represents variable digestive function characterized by fluctuations in digestive capacity. Elevated digestive power represents increased digestive efficiency with relatively rapid digestion. Subdued digestive power represents reduced digestive efficiency with relatively slower digestion.

Study outcomes

The primary outcome was to evaluate the association between Agnibala and serum vitamin B12 status among young adult females.

Secondary outcomes were distribution of the four Agni categories according to vitamin B12 status. Comparison of demographic and anthropometric characteristics between vitamin B12-deficient and vitamin B12-nondeficient participants.

Statistical analysis

All collected data were entered into Microsoft Excel (Redmond, WA, USA), cleaned, and analyzed using STATA statistical software version 10.1 (StataCorp, College Station, TX, USA). Descriptive statistics were used to summarize the study variables. Pearson's chi-square test was used to assess the association between Agnibala categories and vitamin B12 status. The independent Student's t-test was used to compare mean scores for each digestive power domain between the vitamin B12-deficient and vitamin B12-nondeficient groups. Multiple linear regression analysis was performed to identify predictors of serum vitamin B12 levels after adjustment for age and weight. The four digestive strength domain scores were entered into the regression model as continuous quantitative percentage scores rather than as categorical variables; therefore, a categorical reference category was not applicable. A two-tailed p-value ≤ 0.05 was considered statistically significant.

## Results

**Table 1:** Comparison of Demographic, Anthropometric, and Serum Vitamin B12 Characteristics Between Vitamin B12-Deficient and Vitamin B12-Nondeficient Participants Values are presented as mean ± standard deviation (SD). Independent Student's t-test was used to compare continuous variables between the vitamin B12-deficient and vitamin B12-nondeficient groups. The laboratory reference range for serum vitamin B12 was 160–900 pg/mL (140–666 pmol/L) [10]. A p-value ≤0.05 was considered statistically significant.

Variable	Vitamin B12 deficient (n = 47)	Vitamin B12 nondeficient (n = 47)	t-value	p-value
Age (years), mean ± SD	22.83 ± 2.86	22.23 ± 2.74	1.03	0.306
Weight (kg), mean ± SD	51.12 ± 9.19	49.11 ± 7.69	1.16	0.251
Serum vitamin B12 (pg/mL), mean ± SD	118.19 ± 21.62	348.91 ± 94.04	−16.39	<0.001

A total of 94 young adult females aged 19-28 years were included in the study, comprising 47 vitamin B12-deficient and 47 vitamin B12-nondeficient participants. All participants were recruited from the Nagpur region of Maharashtra, India, and followed a vegetarian diet. Baseline demographic, anthropometric, and serum vitamin B12 characteristics of the study participants are presented in Table 1. No significant differences were observed in age or weight between the two groups (p > 0.05). Serum vitamin B12 levels were significantly lower in the vitamin B12-deficient group (118.19 ± 21.62 pg/mL) than in the vitamin B12-nondeficient group (348.91 ± 94.04 pg/mL) (independent Student's t-test, t = −16.392, p < 0.001) (Table 1).

The distribution of dominant digestive strength categories was significantly associated with vitamin B12 status (Pearson's Chi-square test, χ² = 23.16, df = 3, p < 0.001). Among vitamin B12-deficient participants, subdued digestive power was the predominant category (38.30%), whereas balanced digestive power predominated among vitamin B12-nondeficient participants (59.57%). Elevated and irregular digestive power categories showed intermediate distributions in both groups (Table 2).

**Table 2:** Distribution of Dominant Digestive Strength (Agnibala) Categories According to Vitamin B12 Status Values are presented as n (%). Pearson's chi-square test was used to compare the distribution of dominant digestive strength (Agnibala) categories between the two groups (χ² = 23.16, df = 3, p < 0.001). A p-value ≤ 0.05 was considered statistically significant.

Dominant Digestive Strength (Agnibala) Category	Vitamin B12 deficient (n = 47) Number (%)	Vitamin B12 nondeficient (n = 47) Number (%)
Balanced digestive power (Samagni)	6 (12.77)	28 (59.57)
Elevated digestive power (Tikshnagni)	9 (19.15)	3 (6.38)
Irregular digestive power (Vishamagni)	14 (29.79)	9 (19.15)
Subdued digestive power (Mandagni)	18 (38.30)	7 (14.89)
Total	47 (100)	47 (100)

Mean balanced digestive power scores were significantly lower in the vitamin B12-deficient group than in the vitamin B12-nondeficient group (23.40 ± 17.34 vs. 49.70 ± 27.39; t = −5.562, p < 0.001). Conversely, mean elevated digestive power scores were significantly higher in the vitamin B12-deficient group (18.37 ± 24.31 vs. 8.70 ± 16.30; t = 2.265, p = 0.026). No significant differences were observed for irregular or subdued digestive power scores (Table 3).

**Table 3:** Comparison of Mean Digestive Strength Domain Scores Between Vitamin B12-Deficient and Vitamin B12-Nondeficient Participants Values are presented as mean ± standard deviation (SD). Independent Student's t-test was used to compare the mean percentage scores for each digestive power domain between the vitamin B12-deficient and vitamin B12-nondeficient groups. A p-value ≤ 0.05 was considered statistically significant.

Digestive Strength Domain Score (%)	Vitamin B12 deficient (n = 47) Mean ± SD	Vitamin B12 nondeficient (n = 47) Mean ± SD	t-value	p-value
Balanced digestive power	23.40 ± 17.34	49.70 ± 27.39	-5.562	<0.001
Irregular digestive power	28.62 ± 25.78	21.47 ± 24.96	1.367	0.175
Elevated digestive power	18.37 ± 24.31	8.70 ± 16.30	2.265	0.026
Subdued digestive power	29.59 ± 29.21	20.11 ± 20.58	1.818	0.072

Multiple linear regression analysis demonstrated that the overall regression model was statistically significant (R² = 0.26, adjusted R² = 0.21; F(6,87) = 5.00, p = 0.0002). Age and weight were not significant predictors of serum vitamin B12 levels (p > 0.05). In contrast, the digestive strength domain scores showed significant associations with serum vitamin B12 levels in the regression model (Table 4).

**Table 4:** Multiple Linear Regression Analysis of Predictors of Serum Vitamin B12 Levels Values represent regression coefficients (β) obtained from a multiple linear regression model. The four digestive strength domain scores were entered as continuous quantitative percentage scores rather than as categorical variables; therefore, a categorical reference category was not applicable. The model included age, weight, and the four digestive strength domain scores, with an intercept term. The analysis included 94 participants. Model statistics: R² = 0.26; adjusted R² = 0.21; F(6,87) = 5.00; p = 0.0002. A p-value ≤ 0.05 was considered statistically significant.

Predictor of serum vitamin B12 levels	Regression Coefficient (β)	Standard Error	t-value	p-value	95% Confidence Interval
Age (years)	-1.05	4.59	-0.23	0.820	-10.17 to 8.07
Weight (kg)	-1.19	1.55	-0.77	0.440	-4.27 to 1.89
Balanced digestive power score (%)	5248.01	2633.84	1.99	0.050	12.96 to 10483.10
Irregular digestive power score (%)	5246.52	2633.97	1.99	0.050	11.22 to 10481.80
Elevated digestive power score (%)	5245.12	2633.84	1.99	0.050	10.07 to 10480.20
Subdued digestive power score (%)	5245.93	2633.96	1.99	0.050	10.65 to 10481.20

## Discussion

The present study demonstrated a significant association between Agnibala and serum vitamin B12 status among young adult females. Participants with normal serum vitamin B12 levels predominantly exhibited balanced digestive power (Samagni), whereas disturbed digestive power patterns were more frequently observed among vitamin B12-deficient participants. The observed association (χ² = 23.16, df = 3, p < 0.001) suggests that digestive function may influence micronutrient status and provides supportive evidence for the Ayurvedic concept linking digestive efficiency with nutrient assimilation.

According to Ayurvedic principles, Agni is the fundamental force responsible for digestion, metabolism, and the transformation of food into bioavailable nutrients essential for sustaining life. Balanced digestive power facilitates the proper formation of primary nutrient essence (Ahara Rasa), which subsequently nourishes the body tissues (Dhatu) through the sequential actions of elemental metabolism (Bhutagni) and tissue metabolism (Dhatvagni) [13,14]. Thus, digestive power represents the integrated process of digestion, cellular metabolism, and tissue metabolism, whereby food is transformed into a useful component (Prasad) for tissue nourishment and a waste component (Kitta) for elimination [7,12,13]. According to Ayurveda, impairment of digestive power may disturb these metabolic processes, leading to altered digestion, nutrient assimilation, and tissue metabolism [6,15,16].

The present findings suggest that balanced digestive power may be associated with more efficient digestion and nutrient assimilation, as reflected by the higher proportion of participants with normal serum vitamin B12 levels in this category. In contrast, disturbed digestive power patterns, including subdued, irregular, and elevated digestive power, were more common among vitamin B12-deficient participants. According to Ayurvedic principles, subdued digestive power may reduce digestive efficiency, irregular digestive power may produce variable digestive performance, and elevated digestive power may accelerate digestion to an extent that could compromise optimal nutrient utilization [7,12-14,16]. Together, these Ayurvedic concepts provide a plausible explanation for the observed association between digestive strength and serum vitamin B12 status.

According to current physiological evidence, vitamin B12 absorption is a complex process involving coordinated gastric, intestinal, and cellular mechanisms. Following ingestion, vitamin B12 binds to intrinsic factor produced by gastric parietal cells, and the resulting complex is absorbed in the terminal ileum. Disruption of any of these physiological processes, including impaired gastric function, reduced intrinsic factor production, intestinal malabsorption, or altered gastrointestinal motility, may compromise vitamin B12 absorption and contribute to deficiency [17-21]. These physiological mechanisms provide a plausible biomedical explanation for the observed association between digestive function and serum vitamin B12 status in the present study.

The findings of the present study are consistent with the growing evidence that digestive function plays an important role in determining nutritional status. Previous work in women from Eastern Maharashtra has also examined individual physiological constitution (Prakriti) in relation to health-related outcomes [22]. Similarly, studies have demonstrated that gastrointestinal function and dietary factors influence vitamin B12 absorption and micronutrient status [23,24]. In the present study, participants with disturbed digestive power were more likely to have vitamin B12 deficiency, whereas those with balanced digestive power generally exhibited normal serum vitamin B12 levels. Collectively, these findings support the hypothesis that efficient digestive function may contribute to maintaining adequate micronutrient status, although prospective studies are required to establish causal relationships.

Taken together, the present findings integrate classical Ayurvedic concepts with contemporary physiological understanding and suggest that digestive function may be an important factor associated with micronutrient status in young adult females. These findings provide a scientific rationale for further prospective and mechanistic studies to better understand the relationship between digestive strength and micronutrient metabolism.

Strengths and limitations

The present study has several strengths. It is among the first analytical studies to investigate the association between Agnibala and serum vitamin B12 levels using a validated assessment tool together with laboratory-based biochemical estimation. Restricting the study population to vegetarian young adult females reduced dietary and biological variability, thereby improving the internal validity of the findings.

The study also has certain limitations. The relatively small sample size and recruitment from a single geographical region may limit the generalizability of the findings. Furthermore, the cross-sectional design precludes establishing causal relationships between digestive strength and vitamin B12 status. Future multicentre prospective studies with larger sample sizes and comprehensive assessment of dietary intake, gastrointestinal health, and additional biochemical markers are warranted to further validate these findings.

## Conclusions

The present study demonstrated a significant association between digestive strength (Agnibala) and serum vitamin B12 status in young adult females. Balanced digestive power was predominantly observed among participants with normal vitamin B12 levels, whereas disturbed digestive power was more common among vitamin B12-deficient participants. These findings suggest that digestive function may be associated with micronutrient status and provide supportive evidence for the Ayurvedic concept linking digestive efficiency with nutrient assimilation. Further multicentre prospective studies are warranted to validate these findings and elucidate the underlying biological mechanisms.
